# Stakeholder analysis for ‘One Health’ approach to tackle antimicrobial resistance

**DOI:** 10.1136/bmjgh-2025-019236

**Published:** 2025-10-21

**Authors:** Sanjib Adhikari, Komal Raj Rijal, Daniel M Parker, Prakash Ghimire, Phaik Yeong Cheah, Bipin Adhikari

**Affiliations:** 1 Central Department of Microbiology, Tribhuvan University, Kirtipur, Nepal; 2 Department of Population Health and Disease Prevention, Joe C. Wen School of Population and Public Health, University of California, Irvine, California, USA; 3 Department of Epidemiology & Biostatistics, Joe C. Wen School of Population and Public Health, University of California, Irvine, California, USA; 4 Ethical Review Board, Nepal Health Research Council, Kathmandu, Bagmati, Nepal; 5 Center for Tropical Medicine and Global Health, Nuffield Department of Medicine, University of Oxford, Oxford, UK; 6 Mahidol Oxford Tropical Medicine Research Unit, Faculty of Tropical Medicine, Mahidol University, Bangkok, Thailand

**Keywords:** Global Health, Health policies and all other topics, Health systems, Medical microbiology

## Abstract

Antimicrobial resistance (AMR) and interventions to mitigate it are multisectoral, exhibiting super-wicked features that require intersectoral collaboration and synergy. Although AMR and mitigation strategies are pressing issues, their solutions are complex, ethically challenging, multilayered and often conflict at various levels and among diverse stakeholders. The main objective of this study was to identify the values and potential contributions of stakeholder analysis related to AMR and potential interventions from a case study that is being undertaken in Nepal using a ‘One Health’ approach. A total of 33 representatives from human, animal, agricultural and environmental sectors attended a stakeholder meeting in Kathmandu to discuss AMR, its ethical and practical challenges, opportunities and potential interventions. Using a five-pillar framework for stakeholder analysis, we demonstrate its relevance for addressing AMR and propose practical considerations for implementing effective interventions in Nepal. Beyond the practical discussions on AMR and its interventions at the policy, implementation and practice levels, this study underscores the critical value of its methodological reflections for informing ongoing interventions both within Nepal and in similar contexts globally.

**Summary box**

- Antimicrobial resistance (AMR) and interventions to mitigate it bear super wicked features and require multisectoral collaboration and synergy.
- Stakeholder analysis offers an inclusive approach to examine the interests and tensions of multiple sectors ranging from an individual actor to the roles of a health system.
- Knowledge on how AMR intersects through an individual agency, followed by the institution, society and wider actors predicates the success of interventions.
- Stakeholder analysis in AMR using ‘One Health’ approach allows potential for holistic interventions.

## Introduction

 Antimicrobial resistance (AMR) is a pervasive One Health problem that occurs over multiple sectors and at multiple levels.^1^ In this report, ‘antimicrobial’ predominantly refers to antibacterials,^2^ and our focus is on why and how stakeholder engagement is necessary to tackle AMR—a growing threat that, as highlighted by O’Neil’s report, could cause a US$100 trillion economic loss by 2050 and increase AMR-related deaths from 700 000 to 10 million annually if left uncontained.^3^,^5^ Although the problem of AMR has received considerable attention—particularly regarding its identification and burden, including the high-level UN discussion in New York,^6^ the solutions remain grim, rendering AMR a super wicked problem. Part of the reason for AMR is considered super-wicked is its intersectionality, the multipronged nature of its existence, and the ethical dilemmas attached to its solutions.^7^

The ethical dilemmas surrounding AMR are so intertwined that addressing one often exacerbates another, creating a counterintuitive and counterproductive cycle similar to a whack-a-mole problem.^8^,^10^ An ethical analysis of AMR and its interventions identified how restricting the over-the-counter (OTC) use of antimicrobials could actually compromise the access for simple infections such as topical wound infections in remote areas where formal health services are inadequate.^7^ Even selective restrictions of OTC use of antimicrobials can lead to a disorganised landscape of access where policy implementation could suffer from heterogeneity and evasion.^11^ For instance, in Russia, an obligatory 2017 policy that required patients to access antimicrobials only through doctors counterproductively created clandestine and parallel networks evading prescription requirements.^12^ In such scenarios, AMR interventions simply require sensitivity towards social, economic, political and regulatory contexts.^13^ AMR interventions can become futile at the heart when not attuned to the multifactorial impacts they can have.

One of the best-known approaches to dealing with super-wicked problems is through a stakeholder analysis, which fundamentally refers to exploring the interests and landscape of influence surrounding the problem.^14^ This involves a stepwise process of gathering insights on individuals and organisations to assess the resources and influence they bring to the issue, ultimately enabling a clearer understanding of their actions, goals, relationships and interests shaping decision-making and implementation.^14 15^

Interest, position and power are three key factors used for the stakeholder analysis.^15^ Interest pertains to their concern about how a specific policy will impact them and their ability to work effectively. Position indicates their stance—whether they support or oppose the policy. Power, on the other hand, reflects their influence over the policy, determined by their resources and their ability to mobilise them.^16^ Analysing position and interest might be quite straightforward, but with power, it is more complex since it plays a significant role in all processes of policy-making. However, empirical research on the application of disease management policies, particularly in low-income and middle-income countries, frequently fails to adequately characterise power.^17^ In Nepal, the national action plan on AMR was developed in 2016; however, its implementation has been hindered by fragmentation of interests, roles, responsibilities, weak leadership, neglect and constraints in resources.^18^,^20^ Stakeholder mapping has been deemed to unpack some of these challenges embedded at individual, organisational and governance levels. The application of stakeholder analysis has grown across various fields, including environmental management and governance, where businesses, regulators, policymakers and international organisations increasingly rely on it to navigate complex challenges and make informed decisions.^21 22^

Increasingly, a multisectoral approach has been devised to carry out the stakeholder analysis across the disciplines, but the methodologies are heterogenous, undermining their utility at diverse socio-ecological layers.^23^ Stakeholders can be seen as both individuals and representatives of groups or domains of values, beliefs, norms and capacities that intersect with one another, as well as being affected by or influenced by socio-ecological systems. Some of these groups or domains may be more innovative or change friendly than others.^24^ This tool comprises the analyses of stakeholders under five pillars, which reflect the various ways that stakeholders within a system express their agency, roles (both present and future) and influence, all of which work together to highlight overlapping concerns about representation, legitimacy and accountability.^25^ In November 2024, a stakeholder meeting was held in Kathmandu, bringing together 33 representatives—mostly from the policy level—across the human, animal, agricultural and environmental sectors to discuss AMR, including its ethical and practical challenges, opportunities and potential interventions (online supplemental S1 file).

**Figure 1:**
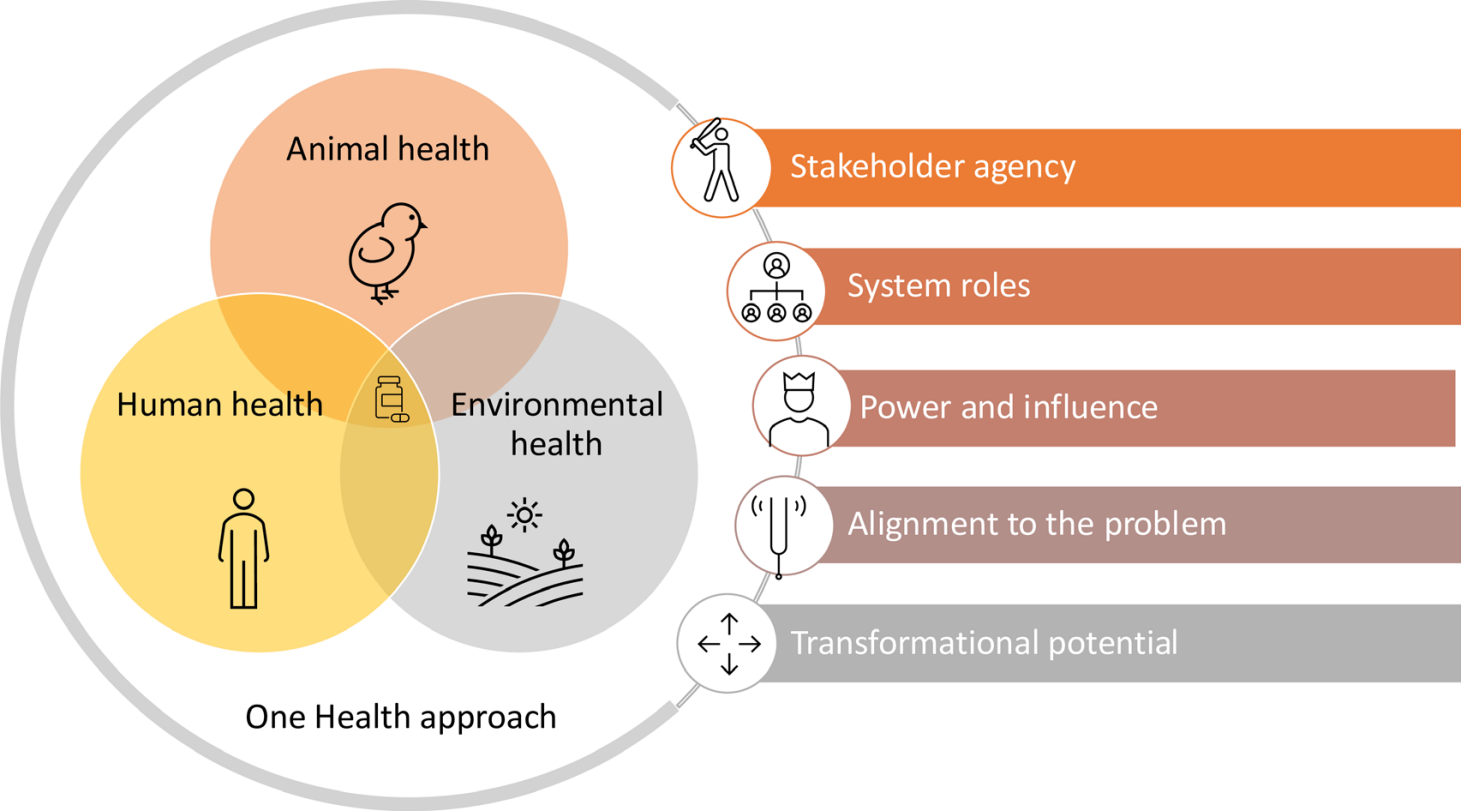
Five components of stakeholder analysis relevant for antimicrobial resistance and its interventions.

In this analysis, we explore the critical role of stakeholder analysis in understanding AMR and its interventions using five key pillars that illustrate how AMR and responses to it are intricately linked, presenting both opportunities and challenges (figure 1).

## Pillar 1: stakeholder agency

Agency is the capacity of individuals and corporate actors to act independently, make choices and exert influence over their circumstances and social structures.^26 27^ Highlighting agency challenges the view of people as vulnerable, emphasising humans as active agents rather than passive responders to environmental (social) adversity.^28^

Agency within stakeholder networks reflects the diverse motivations driving stakeholders’ involvement in knowledge production.^29^ These motivations may not always align with research priorities, necessitating a deeper exploration of stakeholders’ values, needs and underlying goals to foster meaningful collaboration.^30^ Considering stakeholders as agencies instead of just agents renders a plural perspective, allowing capturing their potentials for change. Stakeholders are thus actors with the potential for both acting and becoming.^25^

Antimicrobial consumption is largely governed by the agency of a person or organisation.^31^ For instance, the ability of a person to make autonomous, well-informed decisions about the appropriate use of antibiotics is referred to as their agency. This includes knowing when antibiotics are needed, following treatment correctly and avoiding self-medication or the use of leftover antibiotics.^32^ Cultural beliefs, awareness, access to healthcare and the availability of reliable information all affect an individual’s agency.^33^ Encouraging responsible behaviour and engaging stakeholders about AMR strengthens their role in preventing abuse and makes a substantial contribution to the larger battle against AMR.^34^

Physicians, healthcare workers and drug shops who rely on antimicrobials to treat their patients or serve customers also ultimately serve their livelihood.^7^ Participants discussed how agencies could be multifactorial. For instance, in Nepal, a drug dispenser could be motivated to treat infections while equally motivated to maintain relationship and trust with their customers. Such examples also demonstrated the ethical tensions defying the binary categories of right and wrong. In an institutional environment, the organisational pressure on clinicians to prescribe more and broader class of antimicrobials, for instance, might alter their agency in favour of economic gain and compliance.^31^ Apart from the organisational norms for antimicrobial prescription, clinician’s agency can be further influenced by various other factors, such as diagnostic uncertainty, patients’ demand, time constraints and lack of access to rapid diagnostic tools.^7 31^ Similarly, agencies of people who rely on antimicrobials for livelihood, for instance, drug sellers, pharma companies and government organisation established to regulate antimicrobial use and AMR prevention need further consideration for the proper understanding and management of AMR. Recognising and incorporating agencies from all stakeholders ultimately can inform the tailored and practicable AMR control strategies.^25 31 35 36^ This approach also promotes teamwork and ensures that AMR prevention efforts are comprehensive, lasting and effective, tackling the issue across human, animal and environmental health sectors.^37^

## Pillar 2: system roles

All stakeholders are ultimately linked to a system and their agencies are inextricably connected to and governed by the system roles and norms.^15^ Stakeholder analysis in AMR entails understanding the relationship and interactions within the system. Stakeholders also function as human infrastructural units within the system, amenable to roles, responsibilities and cooperation.^30^ Across the hierarchical spectrum, a policy landscape may be influenced by various priorities. For instance, governments may rely on evidence, healthcare providers on regulations and communities on awareness and access to healthcare. If the system addresses all the stakes at the hierarchy, it will be capable of delivering better results with each role being carried out with higher accuracy.^25^ Operationally, a system may function at multiple categories and both simultaneously and with synchronicity as regulator, decision-maker, guardian, advocate, catalyser/blocker, winner/loser (or beneficiary/disadvantaged) and seller/buyer. In the context of AMR, it is essential to differentiate stakeholders into functional categories to understand their constraints and opportunities for an analysis.^38^

Use of antimicrobials and development of resistance are only revealed through close examination of how each stakeholder’s remit of functional capacity is spanned out.^14^ At different levels, governments, healthcare providers and communities each prioritise evidence, regulation and awareness, respectively. Addressing all these layers strengthens the system’s overall effectiveness.^7^ In the agricultural sector, stakeholders work to minimise misuse of antimicrobials in livestock, while pharmaceutical industries strive to develop novel alternatives.^1 39^ Communities and civil society attempt to promote responsible behaviours, supported by government and non-government organisations. Media and advocacy groups promote awareness and policy implementation. Recognising these system-embedded roles helps identify synergies, gaps and opportunities for an effective AMR response.

Using a ‘One Health’ approach, this stakeholder analysis revealed divergent interests and sector-specific issues in AMR that fail to converge at higher levels, contributing to its macrosocial complexity.^39 40^ In this stakeholder meeting, intersectoral collaboration and synergy were deemed to be a secondary aim to the ongoing primary roles and responsibilities within the sectors. This demonstrated the accountability and roles at each sector dominating the primary remit of functionalities. Any roles requiring coordination and cooperation between three different sectors were beyond the regular scope within the department. This, however, did not underestimate the aims and scope of reaping the benefits from inter-sectoral collaboration. It simply felt a higher ordinance of operationality when adopting the ‘One Health’ approach.

## Pillar 3: power and influence

Power refers to the opportunity (degree of engagement) followed by the capacity of an individual or stakeholders to influence decisions, actions and the distribution of resources that affect their lives and communities.^41^ In the case of AMR and interventions, power refers to the opportunity and leverage of an individual afforded by the role and the position within the system or outside to curb the problem or offer solutions. The power and influence may percolate down the hierarchical spine to devise policies and their implementation.^25^ Power is often distributed as a set of heterogeneous capacity inherent among different actors to influence the goals, processes and outcomes, further intricated by the multigovernance mechanism, such as that of the Federal Republic of Nepal.^42^ Respondents shared that the introduction of three layers of government (federal, provincial and local) has fragmented the governance hierarchy (power and influence), adding new challenges related to coordination and implementation, noting that *‘the new system has also divided the roles and responsibilities vaguely’.* Aligning broadly with the need for an optimisation between ‘governance’ and ‘inclusivity’ for an effective system, proposed by Acemoglu and Robinson^43^; some respondents shared how the tiered health system was affecting the AMR control programmes.

Uneven power dynamics are a fundamental feature of all forms of governance, whether they are monocentric, integrated, decentralised or polycentric.^44^ However, many studies of polycentric governance only offer partial analyses of the initial design or emergent structure of their systems, ignoring or relegating these dynamics to being exogenous to the system.^44^ As a result, several scholars have proposed methods for comprehending the types of power that stakeholders possess in sustainability issues. Power is diverged in three dimensions, that is visible, hidden and invisible and occurring at several levels, for example, at an individual, community and organisation levels operating through closed, claimed and created spaces.^45^ Power is also viewed as innovative, constitutive and transformative operating either in antagonistic or synergistic dynamics.^46^

Exploring this pillar is pivotal in addressing the AMR challenge, as it identifies stakeholders’ capacity to shape decisions, policies and outcomes, while recognising that their power and influence vary depending on the authority they hold over a population. For example, high-power, high-influence stakeholders, such as governments and international organisations, play pivotal roles in creating regulations, funding initiatives and coordinating global efforts.^47^ A stakeholder can also possess dual power depending on their roles within their social sphere. For instance, a person working for a health system that addresses minimising AMR can also be a key actor in a group that promotes antibiotic use.^48^ Similarly, clinicians may hold limited power over patients, but often exert strong influence through trusted medical advice.^31^ Pharmaceutical industries, in contrast, wield broad influence through innovation, though their interests may conflict with economic incentives and regulatory goals.^13^ Despite their distinct power dynamics, veterinary and agricultural industries significantly influence livestock antibiotic use.^1 39^

Contrastingly, even with limited authority, communities can influence AMR policy and implementation through treatment-seeking behaviour and demands for accountability.^49^ Comprehending these power dynamics and their inter-relatedness facilitates focused interventions for an effective response to AMR.

## Pillar 4: alignment

Alignment refers to the possession of stakeholders’ roles and ethical orientations.^25^ Stakeholder alignment analysis focuses on understanding the degree to which stakeholders’ interests, goals and actions align with the overall objectives of addressing AMR. Depending on values placed on the entities such as the environment, people and groups, stakeholders bear a unique set of ideologies about what is morally right or wrong in particular situations.^7 50^ It is critical to acknowledge these moral and ethical orientations for a sustainable transformation of the problem. In addition, navigating through these attributes will allow researchers to identify synergies, manage conflicts and build cohesive strategies across diverse sectors.

Alignment can have different dynamics depending on their moral and ethical duties.^25^ In the case of AMR, these terms can be considered highly subjective and dynamic. For instance, during the discussion, a clinician noted that they would consider it ethically justifiable to use antimicrobials for both therapeutic and prophylactic purposes in a clinic, insofar as such use remains mindful of its cumulative impacts, which could lead to overuse and unnecessary prescriptions—contradicting the recommendation to minimise their use. Thus, a stakeholder may hold multiple moral–ethical stances, including justifications for their behaviour and may not necessarily be motivated by evil intentions.^25^ Stakeholders’ alignment can thus be categorized as actively involved, passively involved, ambivalent, actively opposed or indirectly opposed to AMR programmes targeted for intervention and control.^25^

Alignment in the context of AMR issue evaluates how well stakeholders’ interests, actions and resources resonate with the overall objectives of combating AMR, such as reducing antibiotic misuse, advancing research and promoting stewardship across sectors. This analysis identifies shared goals and potential conflicts, helping to ensure that all efforts complement rather than hinder one another. Stakeholders drive through moral and ethical obligations while working on AMR objectives.^51^ Government or its representatives at a high-level authority responsible for creating and enforcing policies, funding research and implementing national action plans can fail to recognise specific areas where regulatory oversight is critical. For instance, in Nepal, non-binding policies recommend that antibiotics be taken only with a prescription from registered doctors (healthcare workers), but in practice, these regulations misalign with stakeholder interests.^31^

Pharmaceutical and agricultural industries present more complex cases. These sectors align with AMR goals when incentivised to adopt sustainable practices, such as developing antibiotic alternatives or minimising antibiotic use in livestock.^1 13^ However, profit-driven motives, such as promoting broad-spectrum antibiotics, can lead to conflicts with goals of reducing AMR. Similarly, researchers often align closely by generating evidence and innovating solutions, but their work may not always translate effectively into policy or practice.^52^ This analysis will help to navigate the opportunities for the synergies between different stakeholders, such as researchers and policymakers or advocacy groups and communities, and solutions to mitigate misalignment. For example, if public health messaging is inconsistent, policies are not effectively enforced, or financial incentives clash with stewardship initiatives, misalignment may occur. By filling in these gaps, interventions will be more inclusive, well-targeted and better coordinated. This analysis will ultimately result in exploring whether all stakeholders are meaningfully contributing towards the goal of combating AMR.

## Pillar 5: transformation potential

This pillar centres on the ability of stakeholder analysis to drive long-term, systemic change in addressing AMR.^25^ It recognises that AMR is not merely a technical issue but a complex, multifaceted problem that requires a transformation in the way systems interact across human, animal and environmental health sectors.^1 39 47^ A key aspect of this pillar is evaluating stakeholders' readiness to change policies, norms and practices that contribute to AMR dynamics. One key suggestion to transform AMR management across sectors was investing in surveillance at the One Health level—a collective initiative that could lay the foundation for coordinated action across sectors while generating evidence to inform stewardship programmes. Stakeholder analysis helps explore the willingness and ability of people, organisations and systems to initiate and sustain changes that can alter the landscape of AMR. Transformation requires a shift in behaviours, policies and practices, and stakeholder analysis plays a critical role in identifying opportunities for such shifts.^53^ It is, however, critical to pay attention towards how amenable a stakeholder is to the change of policies and functions. Four key properties of transformational readiness have been proposed that are manifested at the level of consciousness, openness, embodying momentum and taking action.^25^ Consciousness refers to stakeholders' awareness of the problems associated with AMR and the need for change. Openness reflects their willingness to embrace new ideas or strategies. Embodying momentum indicates the drive to actively pursue and support change, while taking action signifies the steps stakeholders are willing to take towards implementing solutions. Stakeholder analysis helps identify the areas where change can be most impactful and where interventions can be made to shift behaviours, policies and practices.

## Conclusions

Stakeholder analysis emerges as an important approach in the fight against AMR. By exploring agency, system roles and the power dynamics among stakeholders, it highlights how diverse actors—from policymakers and healthcare providers to communities and industries—interact within complex systems. Understanding these interactions allows for targeted, collaborative interventions that align with stakeholders’ capacities and interests. By leveraging this approach, we can enhance the design and implementation of AMR policies, ensuring that efforts are inclusive, ethical, effective and sustainable across human, animal and environmental health sectors. Applying this five-pillar framework—agency, system roles, power and influence, alignment and transformation potential—can provide a structured analysis of global AMR action plans, enabling the identification of challenges and opportunities for practicable recommendations.

## Supplementary material

10.1136/bmjgh-2025-019236 online supplemental file 1

## Data Availability

All data associated with this article are presented within the article or its online supplemental S1 file.
