# Supplementary material for: Stakeholder analysis for ‘One Health’ approach to tackle antimicrobial resistance
Source: BMJ Glob Health. 2025 Oct 21;10(10):e019236. doi: 10.1136/bmjgh-2025-019236 (PMC12548578; doi:10.1136/bmjgh-2025-019236)
Supplement: online supplemental file 1 [file bmjgh-10-10-s001.pdf]

Supplementary File 1 (S1 File): Stakeholder analysis exercise using one health approach for antimicrobial resistance in Nepal

Sanjib Adhikari[1], Komal Raj Rijal[1], Daniel M Parker[2,3], Prakash Ghimire[1], Phaik Yeong Cheah[4,5], Bipin Adhikari[4,5*]

[1]Central Department of Microbiology, Tribhuvan University, Kirtipur, Nepal

[2]Department of Population Health and Disease Prevention, University of California, Irvine, California, USA

[3]Department of Epidemiology & Biostatistics; Joe C. Wen School of Population and Public Health,

[4]Mahidol-Oxford Tropical Medicine Research Unit, Faculty of Tropical Medicine, Mahidol University, Bangkok, Thailand

[5]Centre for Tropical Medicine and Global Health, Nuffield Department of Medicine, University of Oxford, United Kingdom

*Correspondence: Bipin@tropmedres.ac

AMR-Stakeholder meeting

Date: 13th November 2024

Overview

Stakeholders from animal health, human health and environmental/agricultural health were invited to attend a dinner meeting in Kathmandu on 13th November 2024. A total of 33 delegates attended the meeting representing various divisions and departments of the government from three sectors of the 'One Health'. A broad topic guide related to AMR and intersectoral challenges was discussed among the participants. Based on the discussions, the following major outputs were synthesized using pillars of stakeholder analysis.

Pillars of stakeholder analysis	Sectors	Relevance	Challenges	Opportunities
Stakeholder agency	Human Health	A stakeholder working in the human health sector shared an example of how his efforts in pushing the agenda forward led to discussion and policy incorporation	Roles and responsibilities could change amenable to organizational mandate. Hindrance to the completion of the work started.	Strong agency and intrinsic motivation is critical to trigger policy and implementation while complying with the organizational workflow.
	Animal health	Appreciation of commercial farming and use of antimicrobials was deemed as the foremost step in animal health. Individual agencies seem to have been coordinated through organizational mandate.	Large, coordinated efforts were essential in the animal health sector particularly because of the perturbation and negative ripples likely to arise when implementing stringent rules.	Additional organizational impetus was deemed critical to move forward the agenda of AMR in the animal health sector.
	Environmental health	A large number of pesticides used in the agricultural sector was appreciated but had not achieved adequate attention yet.	The commercial motives of farmers were deemed to be paramount when asked about the actions to curb the use of pesticides.	Increased organizational awareness campaigns and policy recommendations were deemed necessary for future actions.
System roles	Human Health	The development of national action plan 2016 was shared as an	Mere development of national action plan for antimicrobial use	In addition to the national action plan, more

		example of an organizational policy on regulatory principles underpinning the rational use of antimicrobials. Nonetheless, the ethical tensions embedded in the use of antimicrobials were out of scope.	was deemed inadequate mainly because of the systemic governance issues including social, political and economic factors that hindered on its implementation.	resources on its implementation including empirical evidence on antimicrobial regulation and its implications were deemed critical. In addition, disease specific antimicrobial treatment protocols were deemed useful.
	Animal health	Animal health also referred to the national action plan where strategic objectives and activities were deemed to guide the system-based operations against the AMR	Although a clear outline of strategies and activities is laid out, their implementation remains a major challenge mainly as it requires multi-sectoral coordination and concerted efforts.	The need to have concerted efforts have been laid out in the national action plan, which allows stakeholders to follow through and take leads on these activities.
	Environmental health	The use of antimicrobials across the livestock's, feed animals, food products and the environment have been appreciated and the sectoral strategies and activities nonetheless were thought to be inadequate.	The enormity of the sector itself and the multiple stakeholders involved in it pose challenges in coordination and concerted efforts.	Foundational steps in working through increasing awareness on what antimicrobials are and their propensity to cause antimicrobial resistance were considered to be critical.
Power and influence	Human Health	Power and influence stakeholders could pose was very much dependent on the system's premise (the roles and responsibilities). Even if someone at the hierarchical role had high level of power and influence the implementation of policy was deemed to require coordination among multiple	Multi-stakeholders, and fragmentation of power and influence was recognized to be a major challenge. For example, at a clinical setting, a clinician prescribing antimicrobials would be in charge of the decision. At the same time, the chronic implementation challenge	Increased engagement with multiple stakeholders within the human health was being promoted through national action plan including strengthening diagnostic-based infection management.

		sectors and stakeholders (including the end users).	pervaded through the sector in regulating the use of antimicrobials.	
	Animal health	Stakeholders working across the animal health sector recognized the role of power and influence when it came to policy recommendation, and the need for surveillance, but the regular supervision and oversight was poor.	Reconciling the commercial interests of livestock treatment, food animals was deemed difficult because of the wide and heterogenous landscape of farming (e.g. poultry, and fish) that required dedicated resources.	Alternative opportunities for food industry (for example in reducing the use of antimicrobials) was deemed essential. For example, organic farming, market-based promotion of such products.
	Environmental health	Power and influence in the sector of environmental health was deemed more spread and thus required micro-level strategies and engagement.	Structural deficits such as water, sanitation and hygiene were considered to require a systemic effort such as developmental reforms. The use of pesticides for instance in the agricultural sector was considered to be tensed with the commercial interests.	Broad approaches and engagement were thought necessary including starting with the foundational measures of increasing awareness and preventive measures.
Alignment to the problem	Human Health	The problem of AMR was well aligned with the national priority as has been illustrated in national action plan. The alignment seems to be enhanced by the national and international agencies including the discussion on the topic at high level UN meeting, including WHO's support on mitigations strategies to combat AMR.	The multi-layered nature of use of antimicrobials, and development of AMR and their control measures required concerted strategies and efforts.	Increasing engagement with multiple sectors within human health (e.g. clinicians, drug dispensers and patients) and policymakers could enhance the alignment and action towards mitigating AMR.
	Animal health	Priority on use of antimicrobials including implications of AMR development was strengthened by	The historical under-prioritization of animal health sector and rise of antimicrobials' use across food animals was	In addition to renewed interests, and attention, increased resources allocation for surveillance

		the formulation of national action plan.	deemed to be exceeding the threshold for sectoral efforts, particularly as it required resources to monitor the use of antimicrobials.	and monitoring were recognized as opportunities.
	Environmental health	The laid-out problems of environmental health and mitigation strategies in national action plan enhanced the focus and strategies required to supervise and monitor the use of antimicrobials.	The intangibility of the environmental sector where use of antimicrobials has been increasing over the years seem to be the major problem.	Foundational steps in increasing awareness, cleanliness, hygiene and reduction in use of pesticides need to be promoted.
Transformational potential	Human Health	System based regulatory strengthening was deemed critical for example, diagnostic labs, lab-based antimicrobials, mitigation of empirical prescriptions, and prescription audits, including reforming insurance claim-reimbursement mechanisms.	Reconciling the various ethical dilemmas of prioritizing for example, clinical ethics versus public health ethics. Divergence of interests and counter-productive adversities (e.g. access and excess of antimicrobials)	Strengthening laboratory capacities, increasing awareness on responsible use of antimicrobials among all stakeholders.
	Animal health	Resources for research, surveillance and monitoring was considered to be key to transforming the use of antimicrobials and development of AMR.	Enormous commercial interests of food animals. Need to strengthen surveillance and monitoring across a heterogenous landscape of food animals farming.	Increasing surveillance through resource allocation and alternative incentives and management of food industry.
	Environmental health	Promotion of healthy environment, mitigation of pesticide uses in food crops development and multi-pronged and multi-sectoral collaboration	Need for broad and structural reforms. Collaboration across sectors, and regulatory bodies.	Surveillance and promotion of organic farming including community engagement on cleanliness, and hygiene of environment (e.g. water bodies, rivers, greeneries, and healthy living)
